# Potential Fatal Complication of Neurofibromatosis Type 1: Acute Upper Airway Obstruction Due to Ruptured Transverse Cervical Artery Aneurysm

**DOI:** 10.7759/cureus.32910

**Published:** 2022-12-24

**Authors:** Yuji Okazaki, Toshihisa Ichiba, Noritomo Fujisaki

**Affiliations:** 1 Department of Emergency Medicine, Hiroshima City Hiroshima Citizens Hospital, Hiroshima, JPN

**Keywords:** freckling, café-au-lait macules, cutaneous neurofibromas, awake fibrotic intubation, upper airway obstruction, ruptured aneurysm of the branches of the subclavian artery, neurofibromatosis type 1

## Abstract

Neurofibromatosis type 1 (NF1) can cause vascular complications even in undiagnosed NF1 patients. A ruptured aneurysm of the branches of the subclavian artery is a rare but life-threatening event, and the hemorrhage can cause upper airway obstruction. We present a case of NF1 patient with a ruptured transverse cervical artery aneurysm, which led to a nearly obstructed airway. A 52-year-old man who was not previously diagnosed with NF1 presented with sudden pain from the left shoulder to the neck. Since childhood, he has had multiple cutaneous neurofibromas and café-au-lait macules, and freckling in the bilateral axillae. His swollen left side of the neck and left shoulder suggested a hematoma, which compressed the upper airway. Contrast-enhanced computed tomography revealed a cervical hematoma caused by a ruptured aneurysm of the transverse cervical artery. We performed awake fiberoptic intubation because a difficult airway was predicted and surgical airway management may have been impossible due to the anterior cervical hematoma. His airway was secured, and his aneurysm was successfully treated by coil embolization. Based on his cutaneous findings, he was finally diagnosed with NF1. Those who have café-au-lait macules and cutaneous neurofibromas may present with acute cervical hematoma, and it is important to consider the possibility of ruptured aneurysms in the neck region. When patients develop an acute cervical hematoma that causes an acute upper airway obstruction, emergency physicians should consider awake fiberoptic intubation to secure the airway.

## Introduction

Neurofibromatosis type 1 (NF1), also named von Recklinghausen’s disease, is an autosomal dominant disorder that affects the skin, central nervous system, peripheral nervous system, eyes, and bone [1]. Also, in patients with NF1, various-sized arteries can cause stenosis, dissection, and aneurysm [2]. However, some patients may have only cutaneous manifestations from childhood to adulthood. Thus, they do not visit medical institutions for the condition, and their NF1 may remain undiagnosed until the occurrence of potentially fatal bleeding from an arterial rupture.

In patients with NF1, a ruptured aneurysm of a branch of the subclavian artery is a rare but possibly fatal complication [3-6]. The hemorrhage in the neck area is life-threatening because of upper airway obstruction in the narrow pharyngeal space because of compression of the trachea by the cervical hematoma. However, airway management in this unusual circumstance has not been well established in guidelines [7]. In addition, surgical airway management such as cricothyrotomy may be impossible due to an expanding hematoma in the anterior cervical area.

Acute anatomic distortion of the airway requires judicious airway management to prevent total airway obstruction. Rapid sequence intubation (RSI) may be very hazardous in such cases, as neuromuscular blocking agents may interfere with active muscular efforts to keep the airway patent. As a result, the anatomic pathology may make laryngoscopic intubation impossible. In these cases, the best option in appropriately trained hands is awake fiberoptic intubation.

We discuss a patient that presented with undiagnosed NF1 and an acute upper airway obstruction caused by a ruptured aneurysm of the branches of the subclavian artery, in which the airway was successfully secured by awake fiberoptic intubation.

## Case presentation

**Figure 1:**
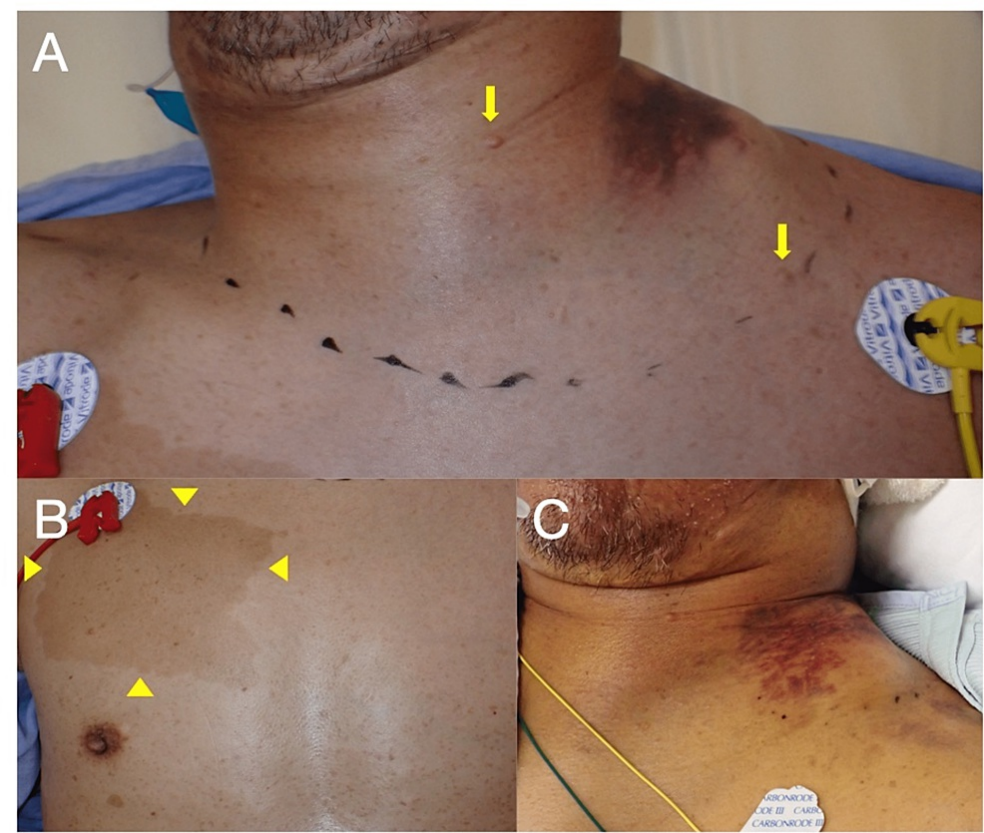
Physical examination. (A) The left side of the neck area was swollen, and the central trachea deviated to the right side. There were many cutaneous neurofibromas in his neck (yellow arrow). (B) Large uniformly hyperpigmented macules suggested café-au-lait macules (yellow arrowhead). (C) The left side of the neck hematoma slightly decreased four days after intensive care unit (ICU) admission.

A 52-year-old man complained of sudden left shoulder pain and swelling around the periclavicular area without a traumatic event. He had a surgical history of acute appendicitis and inguinal hernia, but he had not previously been diagnosed with NF1, although he had hyperpigmented macules since childhood. He did not take any medications. He reported that his mother, who had hyperpigmented macules, died due to ruptured arteries, but she had also not been diagnosed with NF1. On arrival at our emergency department, his vital signs were as follows: blood pressure at the right arm, 202/145 mmHg; heart rate, 120 beats per minute; and oxygen saturation, 97%. Physical examination showed hoarseness and left miosis and ptosis, which suggested the Horner sign. We also found firm and non-pulsatile swelling in the left side of his neck, cutaneous neurofibromas in the neck area (Figure 1A), and multiple uniformly hyperpigmented macules in the anterior thoracic area (Figure 1B) and other body areas, which suggested café-au-lait macules. The café-au-lait macules were not segmentally distributed. The freckling was in bilateral axillae.

**Figure 2:**
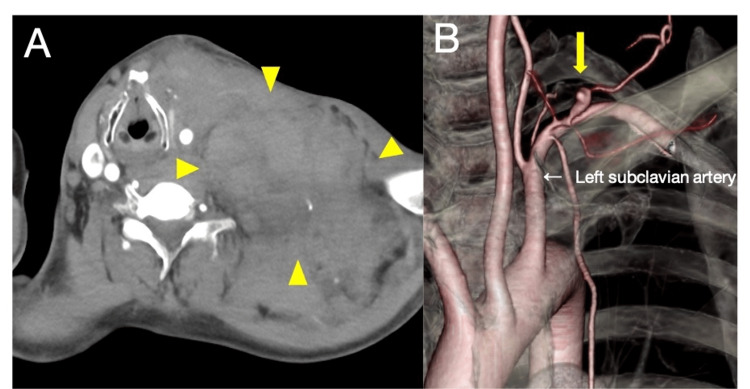
Contrast-enhanced computed tomography. (A) Contrast-enhanced computed tomography (CT) revealed a large hematoma on the left side of the neck area (yellow arrowhead). (B) CT angiography revealed an aneurysm of the branch of the left subclavian artery (i.e., transverse cervical artery) (yellow arrow).

The cutaneous findings had existed since childhood. A laboratory examination showed a hemoglobin level of 16.5 g/dL and normal counts of platelets and coagulation. Contrast-enhanced computed tomography (CT) of the neck and chest revealed extensive hematoma from the neck to the mediastinum, which compressed the upper airway from the left side (Figure 2A), resulting from a ruptured aneurysm of the branch of the subclavian artery (Figure 2B).

**Figure 3:**
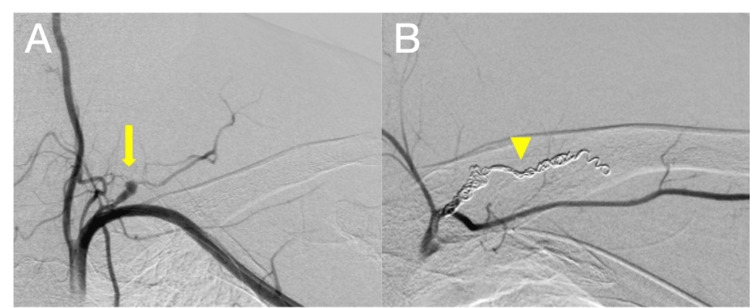
Angiography (A) Angiography revealed a transverse cervical artery aneurysm without extravasation (yellow arrow). (B) We successfully performed coil embolization for the aneurysm of the artery (yellow arrowhead).

While in our department, the hematoma of the neck increased, and he developed severe dyspnea, although he retained a normal level of consciousness. Impending upper airway obstruction was suspected, and urgent airway protection was indicated. His airway was deviated due to a hematoma that extended to the anterior neck, and we predicted that his supraglottic space would further narrow. Because of his cooperation, we chose transoral awake tracheal intubation using flexible bronchoscopy to protect his airway. Morphine 10 mg was intravenously administrated, and topical anesthesia was performed with lidocaine 8% spray. His airway was successfully protected using a 7-mm endotracheal tube with good patient tolerance. After he was sedated with propofol, coil embolization was performed for the ruptured aneurysm of a branch of the subclavian artery (Figures 3A, 3B). He was finally diagnosed as having a ruptured transverse cervical artery aneurysm.

After coil embolization, he was admitted to the intensive care unit (ICU). The hematoma from the neck to the shoulder had not extended, and his hemoglobin level did not decrease. He was treated with rest and airway protection. Four days after ICU admission, his neck hematoma had slightly decreased (Figure 1C). Fortunately, a contrast-enhanced CT at eight days after onset revealed that the hematoma had slightly reduced, and laryngoscopy did not reveal edema of the vocal cord and supraglottic space. He was successfully extubated in the operating room nine days after onset, with preparation to perform an emergency reintubation or tracheostomy as required. Whole-body CT did not reveal other arterial aneurysms, including the brain, central nervous system neoplasms, bone abnormalities such as long bone dysplasia and scoliosis, or soft tissue sarcomas. We diagnosed this case as NF1 based on revised diagnostic criteria for NF1 [8]. The Horner sign (i.e., miosis and ptosis) and his left swollen neck and shoulder gradually improved, and seventeen days after onset, he was discharged without any complications.

## Discussion

The course of this patient indicated two important clinical issues: (1) undiagnosed NF1 patients may present with unusual but potentially fatal vascular complications, such as a ruptured aneurysm of the branch of the subclavian artery, and (2) in cases of acute cervical hematoma that threaten acute upper airway obstruction, awake fiberoptic intubation is the preferred choice of airway management in the emergency department.

Patients with NF1 may have unusual vascular complications, such as a ruptured aneurysm of the branch of the subclavian artery [3-6]. Vascular complications in NF1 patients typically include the renal artery, aorta, and cerebral and vertebral arteries. However, the peripheral arteries (i.e., tibial artery [9] or ulnar artery [10]) and external carotid artery [11] rarely rupture but can be fatal in patients with NF1. Thus, if patients with café-au-lait macules and cutaneous neurofibromas, even without a diagnosis of NF1, develop unusual hemorrhage or swelling, vascular complications of NF1 should be taken into account.

Fatal upper airway obstruction caused by an acute cervical hematoma is one of the important causes of mortality and morbidity in patients with NF1 [3,12], while malignant neoplasms in NF1 are also common causes [13]. The pharyngeal and supraglottic spaces and trachea were externally compressed due to the expanding acute hematoma of the neck region, which may lead to be deviation and narrowing of the airway and lead to severe dyspnea. In this case, after admission to our emergency department, there were approximately thirty minutes before severe dyspnea occurred, but the hematoma caused by ruptured aneurysms could be life-threatening within a few minutes after onset [12]. Thus, in patients with acute cervical hematoma, we should consider prompt securing of airway management, including the use of flexible bronchoscopy, before the occurrence of critical respiratory distress.

Awake fiberoptic intubation is likely to be the preferred choice of airway management in patients with an extremely compressed and narrowed upper airway caused by an acute cervical hematoma [7]. Although various types of devices (e.g., video-laryngoscopy with a hyperangulated blade or gum-elastic bougie) have been developed for difficult airways [14], management in patients with upper airway obstruction still remains challenging. Surgical airway protection (i.e., the insertion of a front-of-neck airway) is one other option, but it may be difficult with an expanding hematoma to the anterior neck. In contrast, using fiberoptic intubation is an effective technique for airway management in such a situation. It is dangerous to perform RSI in situations where a difficult airway is anticipated. Thus, if the patient is able to cooperate, awake intubation should be considered [14]. The choice between awake laryngoscopic intubation and awake fiberoptic intubation depends on the skills and tools available. Although there have been studies of awake fiberoptic intubation before scheduled surgery in operating rooms [7], there have been few studies of emergent awake fiberoptic intubation in the emergency department. Although a rare situation, it is necessary for emergency physicians to attain competency and maintain skills in awake fiberoptic intubation and for emergency departments to have the necessary equipment available.

## Conclusions

Even in patients without a diagnosis of NF1, those who have café-au-lait macules and cutaneous neurofibromas may present with acute cervical hematoma, and it is important to consider the possibility of a ruptured aneurysm in the neck region. In such cases, emergency physicians need to promptly secure the airway under spontaneous breath with flexible bronchoscopy.
